# Developmental trajectories and breakdown in F1 interpopulation hybrids of *Tribolium castaneum*

**DOI:** 10.1002/ece3.536

**Published:** 2013-05-24

**Authors:** Douglas W Drury, Ross C Ehmke, Victoria N Jideonwo, Michael J Wade

**Affiliations:** Department of Biology, Indiana UniversityBloomington, IN

**Keywords:** Development, hybridization, speciation, Tribolium

## Abstract

When hybrid inviability is an indirect by-product of local adaptation, we expect its degree of severity between pairs of populations to vary and to be sensitive to the environment. While complete reciprocal hybrid inviability is the outcome of the gradual process of local adaptation, it is not representative of the process of accumulation of incompatibility. In the flour beetle, *Tribolium castaneum*, some pairs of populations exhibit complete, reciprocal F1 hybrid incompatibility while other pairs are fully or partially compatible. We characterize this naturally occurring variation in the degree and timing of expression of the hybrid incompatible phenotype to better understand the number of genes or developmental processes contributing to speciation. We assessed the morphological and developmental variation in four *Tribolium castaneum* populations and their 12 possible F1 hybrids at each life-history stage from egg to adult. We find that the rate of hybrid larval development is affected in all interpopulation crosses, including those eventually producing viable, fertile adults. Hybrid incompatibility manifests early in development as changes in the duration of instars and diminished success in the transition between instars are relative to the parent populations. Parent populations with similar developmental profiles may produce hybrids with disrupted development. The degree and timing of expression of hybrid inviability depends upon populations crossed, direction of the cross, and environment in which hybrids are raised. Our findings suggest that the coordinated expression of genes involved in transitional periods of development is the underlying cause of hybrid incompatibility in this species.

## Introduction

Previous work by Demuth and Wade (2007a,b) discovered widespread adult hybrid incompatibility among populations of the red flour beetle, *T. castaneum*: 3% to 100% of adult interpopulation hybrids exhibit negative phenotypes such as deformities of the limbs, antennae, mouthparts, and wings. Quantitative genetic analyses revealed that these had a complex genetic architecture with maternal genetic effects and Genotype-by-Environment interactions. Some interpopulation crosses exhibited Haldane's Rule (Demuth and Wade 2007b), wherein the heterogametic male hybrids exhibited more frequent deformities than their homogametic hybrid sisters. The manifestation of this classic interspecific pattern in the interpopulation hybrids suggests that its origin lies early in the speciation process. In the Bateson-Dobzhansky-Muller (BDM) speciation model, genes with a positive fitness effect in the sympatric background but a negative effect in a hybrid background cause reproductive incompatibilities. Thus, the variation observed within the *T. castaneum* species for interpopulation compatibility represents the “polymorphic prelude to BDM incompatibilities” (Cutter 2012), affording an opportunity to dissect the genetic causes of incompatibility early in the speciation process. These data also support the view that hybrid incompatibility is an indirect by-product of adaptation to local environments, with some population pairs more divergent, and therefore more incompatible, than others.

Drury et al. (2011) mapped an extreme interpopulation incompatibility, the complete absence of F1 adults, to a region in linkage disequilibrium with the *T. castaneum* ecdysone receptor (*EcR*) homolog. *EcR* is a major regulatory switch, with multiple alternative splice forms; it is known to control larval molts, time of pupation, and eclosion (Li and Bender 2000). Although such extreme incompatibilities, with their alternative phenotypes, are easier to map, they represent the final stage in an underlying continuous process when hybrid inviability evolves as an indirect by-product of adaptation to local environments (Rice 1987). However, other population pairs manifesting partial incompatibility have received much less genetic attention.

Larval development and the transition from pupae to adult is a complex trait, especially in the holometabolous insects. It has long been speculated that small differences in developmental timing, known as heterochrony, might play an important role in speciation (McMillan et al. 1992; Mabee et al. 2000). The central idea is that a small difference in the timing of critical events during development may lead to larger, corresponding changes in adult morphology and thus contribute to rapid speciation (e.g., Alberch et al. 1979; Alberch 1980; Kamiya 1992). Our findings with regard to speciation and heterochrony are somewhat different. The adult and larval morphologies in our populations of *T. castaneum* are indistinguishable. However, the underlying genetic mechanisms for the timing developmental events during maturation and their sensitivity to density and temperature have become sufficiently different among populations to cause partial or complete developmental failure of the immatures.

Interactions between the maternal and zygotic genomes are critical to the control of events in early development and are known to diversify rapidly in fruit flies (Cruickshank and Wade 2008). Demuth and Wade (2007a,b) in quantitative genetic studies of adult hybrid deformities in *T. castaneum* found evidence of an important maternal genetic component. Here, we have extended those studies to the timing of hybrid larval development. Surprisingly, populations with similar patterns of larval development may produce larval hybrids whose development is different from that of either parent. This pattern suggests that local selection affects larval development and that small differences in timing can become the basis for reproductive isolation.

In contrast to studies of interpopulation adult hybrids (Demuth and Wade 2007a,b; Drury and Wade 2011; Drury et al. 2011), the earlier developmental stages, larvae and pupae, have not been characterized in interpopulation crosses. Because small errors early in development can be compounded during ontogeny (Arthur 2004), it is likely that the majority of hybrid adult developmental deficiencies result from earlier developmental deficiencies of the immatures. We investigated hybrid larvae from a factorial combination of crosses between four populations to determine the frequency of larval developmental deficiencies and their relationship to adult hybrid deformities. Earlier detection and better classification of hybrid abnormalities, in all developmental stages, can speed the characterization, differentiation, and mapping of nascent incompatibilities.

We made all possible reciprocal crosses among four populations from India, South America, Canada, and Georgia (U.S.A.). Eggs from each cross were reared at a controlled, constant density, as well as at a natural density achieved by 24 hours of egg laying. Eggs at each density were also raised at two temperatures, 26 and 34°C, the rearing temperatures of the hybrid adults characterized by Demuth and Wade (2007a,b). At 4-day intervals, we censused each set of developing eggs or larvae, photographed them through a stereomicroscope, and measured larval length from the stored digital images. We used these measurements to estimate a series of developmental parameters, including growth rates, asymptotic larval size, and size at adulthood as functions of temperature and life stage (egg, larval instar, pupa, adult). We also report larval to adult viabilities. We compared the developmental trajectories from egg to adult of interpopulation hybrids with those of the pure-bred parent populations to determine the extent of hybrid developmental dysfunction and its stage specificity.

## Methods

### Experimental design

We used four populations in this study. The Georgia (U.S.A.) stock, GA-1, is a standard laboratory strain, used in sequencing the *T. castaneum* genome (Richards et al. 2008). The Colombian (*ab*) and Indian (Raj-1) strains were wild collected and have been reared in the laboratory at large-population size for over twenty years. The Colombian population expresses a maternal-effect selfish phenotype, MEDEA (Lorenzen et al. 2008). Various hybrid-dysfunctions have been reported in crosses between Indian populations and MEDEA containing populations (Thomson et al. 1995; Thomson and Beeman 1999). The Canadian population is an independently derived MEDEA-expressing population which is phenotypically distinct in respect to hybrid-dysfunction.

We set up a total of 16 different F_1_ crosses factorially combined with two rearing temperatures and two densities (4 populations × 4 populations = 16 crosses; two temperatures and two densities for a total of 16 crosses × 4 environments = 64 treatments). For each cross, we allowed 20 pairs of parents, to lay eggs for 24 hours at 30°C. The eggs laid during the first 24-hour time interval were split into two equally sized groups (48 eggs each) and placed in 10 g of standard medium (20:1, flour:brewer's yeast, by weight). One group was reared at 34°C while the other was reared at 26°C. All eggs produced in a second 24-hour laying period were also split into two groups and also raised at the two different temperatures. This protocol produced four groups of evenly aged eggs from each pair of adults, distributed across two egg-density treatments: (1) a controlled-density treatment with exactly 48 eggs; and, (2) an uncontrolled-density treatment. Larvae from every rearing condition and cross were sifted from the medium, cold anesthetized and photographed every 4 days throughout development. Photographs were taken with a Canon EOS Rebel XS Digital SLR Camera (Tokyo, Japan) with Canon EF-S 18–55 mm IS lens mounted on a Zeiss Stemi 2000-C stereomicroscope. Photos were analyzed in ImageJ, where measurements were converted to millimeters (mm).

### Estimating growth parameters

Growth curves reveal time-dependent nonlinear changes of the body or organ measurements in organisms and can be used to predict the expected size of a group of animals at a specific time points. We fit our measurements of larval length to the Gompertz growth model (Gompertz 1825; Wang and Zuidhof 2004):


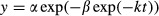


where *y* is the observed length at measurement time *t*, the age of the sample. The three parameters estimated using the Gompertz function are *α*_,_ size at maturity, *β*, the logarithm of the ratio of mature size to initial or hatching size, and *k*, the maturation rate.

Growth in holometabolist insects occurs only during the pre-pupal stages. Thus, α is the predicted larval size when growth has finished. Thus, the most informative growth parameters are the size at maturation (α) and maturation rate (*k*). Growth parameters have been shown to be heritable and can be modified by both natural and artificial selection (Hardin and Bell 1967; Roff 2000). The nonlinear least-square regressions were performed with “nls” function in the R statistical environment (R Core Development Team 2008). We estimated the growth parameters of each treatment (64) separately, along with the 95% confidence interval (Tables 1 and 2) of both parameters.

**Table 1:** Estimates of larval asymptotic size (α) for each of the 64 treatments

	Dam type							
	Low temperature				High temperature			
Sire type	Canada	Colombia	Georgia	India	Canada	Colombia	Georgia	India
Uncontrolled density								
Canada	6.03 (5.59–6.47)	6.67 (6.15–7.19)	6.08 (5.97–6.20)	6.12 (5.78–6.12)	5.69 (4.64–6.75)	4.74 (4.40–5.08)	6.70 (6.37–7.03)	6.12 (5.65–6.59)
Colombia	6.76 (5.91–7.60)	7.33 (6.51–8.16)	6.81 (6.64–6.98)	inviable	5.82 (4.89–6.66)	7.87 (7.32–8.42)	5.82 (5.41–6.22)	inviable
Georgia	6.30 (5.82–6.78)	7.01 (6.81–7.22)	6.93 (6.65–7.20)	6.74 (6.61–6.87)	6.89 (5.86–8.09)	6.20 (5.40–7.00)	6.09 (5.97–6.20)	6.35 (6.16–6.54)
India	6.00 (4.48–7.51)	inviable	6.13 (5.80–6.46)	7.74 (7.26–8.22)	6.37 (4.37–8.36)	2.72 (2.59–2.84)	6.41 (6.01–6.82)	5.95 (5.82–6.08)
Controlled density								
Canada	5.51 (4.88–6.13)	*	6.55 (6.06–7.03)	5.32 (5.19–5.45)	5.62 (5.08–6.16)	*	5.37 (5.10–5.65)	5.23 (4.93–5.53)
Colombia	*	6.39 (6.16–6.62)	7.08 (6.49–7.68)	inviable	*	5.98 (5.60–6.36)	5.59 (5.30–5.89)	inviable
Georgia	4.85 (2.52–7.18)	6.34 (6.00–6.67)	5.92 (5.25–6.58)	7.24 (6.49–8.00)	7.16 (1.19–13.12)	6.36 (5.70–7.02)	5.43 (3.98–6.88)	5.12 (4.73–5.52)
India	5.55 (4.85–6.25)	inviable	8.51 (6.16–10.86)	6.55 (6.23–6.87)	5.59 (4.58–6.59)	3.03 (2.81–3.25)	6.46 (5.97–6.95)	5.77 (5.35–6.19)

All estimates correspond to millimeters (mm).

* designates insufficient biological samples for the model to achieve convergence, low temperature: 26°C, high temperature: 34°C.

**Table 2:** Estimates of larval growth rate (*k*) for each of the 64 treatments

	Dam type							
	Low temperature				High temperature			
Sire type	Canada	Colombia	Georgia	India	Canada	Colombia	Georgia	India
Uncontrolled density								
Canada	0.064 (0.053–0.074)	0.070 (0.060–0.081)	0.090 (0.085–0.095)	0.065 (0.061–0.069)	0.137 (0.103–0.171)	0.221 (0.186–0.256)	0.134 (0.124–0.144)	0.141 (0.125–0.156)
Colombia	0.075 (0.057–0.092)	0.049 (0.042–0.057)	0.074 (0.070–0.078)	inviable	0.273 (0.177–0.369)	0.103 (0.092–0.114)	0.184 (0.161–0.207)	inviable
Georgia	0.086 (0.072–0.099)	0.075 (0.071–0.080)	0.054 (0.05–0.058)	0.082 (0.077–0.087)	0.151 (0.116–0.186)	0.154 (0.128–0.181)	0.147 (0.138–0.156)	0.151 (0.142–0.161)
India	0.043 (0.026–0.060)	inviable	0.082 (0.072–0.092)	0.056 (0.051–0.060)	0.119 (0.079–0.159)	0.192 (0.153–0.231)	0.133 (0.118–0.148)	0.172 (0.162–0.183)
Controlled density								
Canada	0.094 (0.073–0.114)	*	0.089 (0.075–0.104)	0.107 (0.098–0.117)	0.155 (0.129–0.182)	*	0.313 (0.233–0.393)	0.294 (0.241–0.347)
Colombia	*	0.081 (0.074–0.089)	0.087 (0.074–0.100)	inviable	*	0.223 (0.175–0.271)	0.324 (0.254–0.394)	inviable
Georgia	0.126 (0.033–0.219)	0.101 (0.089–0.114)	0.084 (0.068–0.099)	0.087 (0.073–0.101)	0.157 (0.020–0.294)	0.264 (0.191–0.337)	0.211 (0.111–0.311)	0.379 (0.271–0.487)
India	0.057 (0.042–0.072)	inviable	0.046 (0.034–0.059)	0.080 (0.072–0.089)	0.272 (0.132–0.412)	0.137 (−0.035–0.309)	0.188 (0.165–0.210)	0.216 (0.165–0.268)

All estimates correspond to millimeters per day (mm*day^−1^)

* designates insufficient sample size for the model to achieve convergence; low temperature: 26°C, high temperature: 34°C.

### Other statistical tests

To assess the factors contributing to differences in larval to adult viability we used a hierarchical *G*-test of heterogeneity. Preliminary tests showed no effects of density on survival or on deformity frequency. Therefore, we divided the survival data set into eight clusters to investigate heterogeneity of the data: parental crosses within temperatures, parental crosses across temperatures, hybrid crosses within temperatures, hybrid crosses across temperatures, and all crosses across all temperatures.

## Results

### General results

The summary statistics for the estimates (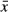, and 95% CI) of asymptotic larval size (α) and growth rate (*k*) are presented in Tables 1 and 2. We were unable to estimate the parameters of the growth model for several of the hybrid crosses. The four controlled-density treatments (two reciprocal crosses at two temperatures) of the Colombia dam-by-Canada sire and Canada dam-by- Colombia sire crosses were excluded due to very low egg-to-first instar larval viability. The number of larvae reaching the final stages of development in these treatments was prohibitively low for the model fitting procedure to obtain convergence. Six other treatments were also excluded from statistical analysis due to complete egg or larval inviability within the first 8 days of development. These treatments included the Colombia dam-by-India sire crosses reared at 26°C and all treatments involving the reciprocal cross. Each of these ten treatments shows some degree of incompatibility affecting early development in crosses among our four populations. In addition, larvae in two of our crosses failed to pupate (see below). Thus, 25% of our interpopulation crosses (12 of 48) exhibited some degree of hybrid infertility (postzygotic, prehatching) or inviability (larval mortality post hatching).

Temperature manipulation influenced growth rate, *k*, as expected; growth rate estimates were significantly lower in the colder, 26°C treatments than in the warmer treatments (*t*-test: *t* = 8.3822, df = 52, *P* < 0.001). Furthermore, the predicted asymptotic size of larvae from the 26°C treatments was significantly larger than larvae from the warmer treatment. The mean final size (α) of cold reared larvae is 6.4792 mm (SEM = 0.1528) while the mean of the larvae reared at 34°C is 6.0269 mm (SEM = 0.1319) (*t*-test: *t* = 2.2405, df = 50, *P* = 0.0295). Two treatments where larvae did not reach pupation (Indian sire-by-Colombian dam at 34°C) were removed from calculations of these parameters.

To determine if the temperature differences in predicted larval size persisted through the pupal stage to adult metamorphosis, we recorded size data for adults from the same population. The influence of rearing temperature on adult size was comparable to that from the larval data. Adults which developed at lower temperatures were larger (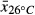 = 3.685 mm, SEM = 0.0079; 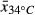 = 3.657 mm, SEM = 0.0081; *t* = 2.4890, df = 1767, *P* = 0.0129).

### Population growth curves

#### Growth rate: parental strains

We compared our populations for asymptotic size and growth rate, parameters of the Gompertz equation, to determine if hybrids differ from their parent populations or if they respond differently from them to temperature and density.

We consider populations reared at 34°C and controlled densities as the standard conditions, since these environmental conditions produce the fewest abnormalities (see below). Furthermore, all four parent populations have statistically indistinguishable growth rates when reared at these conditions (Colombia: 0.223 mm/day, 95% CI: 0.175–0.271; Georgia: 0.211 mm/day, 95% CI: 0.111–0.310; Canada 0.156 mm/day, 95% CI: 0.129–0.182; India: 0.216 mm/day, 95% CI: 0.165–0.268; no pair-wise comparisons were significant *P* > 0.05). When individuals from the same set of parents are reared in the colder environment, the growth rates are significantly reduced by 57% to 80%. Although the reduction in growth rate at the colder temperature is not significant for the Canada population, the proportional reduction of 57% is comparable to that of Georgia (62%) and Colombia (58%) (Canada: 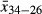 = 0.0627 mm/day, *P* = >0.05; Colombia 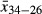 = 0.1297 mm/day, *P* < 0.0001; Georgia 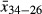 = 0.0961 mm/day, *P* = 0.0469; India 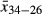 = 0.136, *P* < 0.0001).

We observed no significant effect of density on growth rate (*k*) at our control temperature (34°C) (all comparisons *P* > 0.05). However, at the colder temperature (26°C), some of the changes observed in growth rate between low and high density are significant. The Canada and Colombia populations have lower growth rates at the higher density but the differences were not significant (Colombia 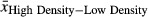 = −0.0126 mm/day, *P* >0.05; Canada 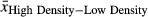 = −0.0335 mm/day, *P* > 0.05). However, the Georgia and India beetles grew significantly more slowly at the higher density at cold temperature (Georgia 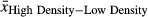 = −0.0696 mm/day, *P* < 0.0001; India 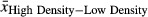 = −0.0452 mm/day, *P* < 0.0085). The relative magnitude of the effect of density on growth rate (21% to 33%) was smaller than the differences in growth rates caused by the reduction in temperature.

### Differences among hybrid crosses

We investigated all possible out-cross types to determine if, hybrid growth rates differ from the paternal type, maternal type, or from the reciprocal F_1_. First, we tested whether the mean hybrid of a given cross deviated from the parental midpoint: 64% (23/36) of hybrid crosses exhibited a significant deviation from the parental midpoint. These deviations showed clear directionality in that all but two (21 of the 23 significant differences) had a faster mean growth rate than the corresponding mid-parent value (deviation from the random expectation: *χ*^*2*^_1_ = 15.6957, *P* < 0.001). Direct comparisons of growth rates of the reciprocal hybrids with that of the paternal or maternal parent tend to show an interaction across treatments. For example, in the low-density Colombia-by-Georgia crosses, at 34°C, the hybrid offspring had growth rates slower than that of the paternal parent (difference = −0.0794 mm/day, *P* = 0.001) but, at 26°C, hybrid growth rate was significantly faster than that of the paternal strain (diff = 0.0641 mm/day, p < 0.001). Hybrids from the India sire-by-Canada dam cross developed significantly faster than either parental type at high density and high temperature (a difference of 0.0657 mm/day, *P* < 0.001). No systematic hybrid larval deformities of the exoskeleton were detected other than those previously described in Drury et al. (2011). However, in one cross, we did find a systematic developmental dysfunction. In the Canada x Indian crosses at 26°C, a majority of individuals do not successfully eclose during the transition from pupa to adult. This leaves individuals trapped in the pupal case and effectively inviable.

### Asymptotic larval size

The asymptotic or final larval size did not differ among parent types at standard conditions. However, both the Colombia and India strains significantly increased in larval size when reared in colder environments, with the Colombia mean length, summed across density treatments, increasing 6.8%, from 5.982 mm to 6.388 mm (*P* < 0.001) and the India larvae increasing 13.6%, from 5.767 mm to 6.550 mm (*P* < 0.001). Size in the Georgia and Canada populations did not change significantly with temperature. Two hybrid combinations, India sires-by-Georgia dams at high densities and India sires-by-Canada dams at low temperatures and high densities, showed differences in asymptotic larval size. There were no differences observed between reciprocal crosses owing to density.

### Survival

**Figure 1:**
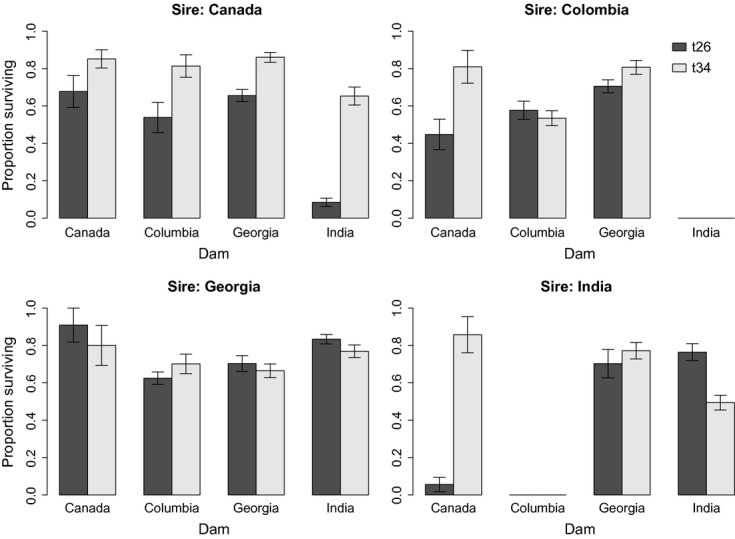
The portion of larvae which reach free-living adults. Bars display the mean (± SEM) survival, paired by temperature with dam nested within sires. All offspring of the Indian sires-by-Colombian dam and Colombian sire-by-Indian dam, and both temperatures do not reach adulthood.

Survival of parents and their interpopulation hybrids was assayed in two stages: (1) survival from egg to larva; and, (2) survival from early larva to adulthood (Fig. 1).

Tribolium beetles have the ability to lay unfertilized eggs when not inseminated, although the rate of egg lay is very low (Lopez-Fanjul and Jodar 1977). These eggs do not hatch and desiccate within the first few days after laying. Although we have no estimate of the proportion of fertilized to unfertilized eggs, all females in our treatments were inseminated and laid abundant eggs, so that our measurement of egg-to-larval viability is minimally affected by unfertilized eggs not fated for hatching. Egg-to-larval survival among the parental populations at control conditions ranged from a low of only 19% [SEM: 0.057] for the Georgia population to a high of 73% egg-to-larva survival in the Colombia population. The parent populations differed significantly from one another in average egg-to-larva survival across all conditions, with the Georgia strain having the lowest overall survival, 25% [SEM 0.044] and Columbia the highest, 75% [SEM 0.044]. Little of this effect was owing to temperature; at the colder treatments, survival was lowered only in the Canada population (Canadian, *χ*^*2*^_1_ = 13.44, *P* = 0.0002; Colombia, *χ*^*2*^_1_ = 0.056, *P* = 0.814; Georgia, *χ*^*2*^_1_ = 1.389, *P* = 0.2385, India, *χ*^*2*^_1_ = 1.043, *P* = 0.3070). The variance in survival among the hybrid crosses was much greater than that observed among the parental crosses. In the hybrids, egg-to-larval survival ranged from 0% in the Colombia dam-by-Canada sire cross to 83% [SEM 0.071] in eggs produced by the India dam-by-Canada sire cross.

Survival from larva to adult did not vary among parental crosses at 26°C (Fig. 1; *G*-value = 3.093, df = 7, *P* = .8707) or at 34°C (*G*-value = 9.684, df = 7, *P* = 0.2225). Across all hybrid crosses, the survival from early larvae to adulthood did not vary when reared at 34°C (Table 3; *G*-value = 6.999, df = 19, *P* = 0.9943). However, when reared at 26°C substantial heterogeneity in survival rates exists among hybrid cross types (*G*-value = 116.36, df = 19, *P* < 0.0001). (Note that these statistics exclude crosses already identified as completely inviable as eggs or early larvae.)

**Table 3:** *G*-test for heterogeneity of survival rates from 4-day larvae to adult

*G*-test	*G*-Value	Df	*P*-value
Parental 26°C	3.093	7	0.8707
Parental 34°C	9.684	7	0.2225
Pooled parental	13.876	15	0.5552
Hybrids 26°C	116.357	18	<0.0001
Hybrids 34°C	6.99	19	0.9943
Hybrids pooled	138.19	38	<0.0001
Total pooled	153.17	54	<0.0001

### Deformities

**Figure 2:**
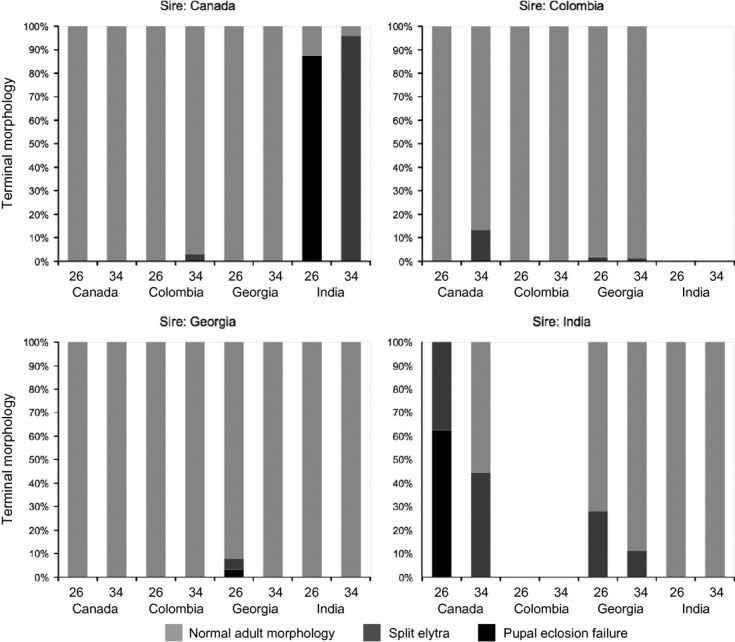
The frequency of terminal abnormalities among crosses. All offspring of the Indian sires-by-Colombian dam and Colombian sire-by-Indian dam do not reach adulthood and are thus excluded. Figure 3 provides a phenotypic description and illustration of the three morphologies.

**Figure 3:**
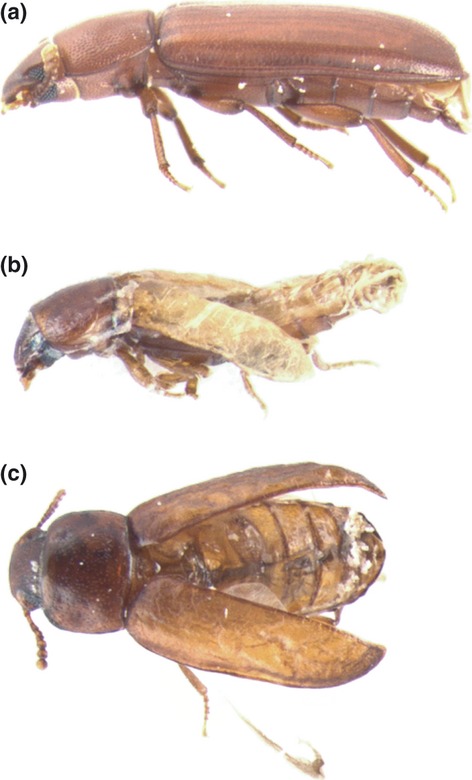
(a) Lateral view of normal adult beetle morphology. (b) Representative hybrid beetle of an Indian sire-by-Canada dam cross-reared at 26°C. These beetles die due a failure to complete eclosion. (c) Representative hybrid beetle with split elytra (Indian sire-by-Canada dam cross-reared at 34°C) [Photo by: Franck Simonnet.]

Temperature had the largest effect on survival on Canada-by-India hybrids. When the maternal parent is of Indian origin and the larvae are reared at the colder temperature, only 12.5% of larvae survive to free-living adults. The other 87.5% do not complete the transition from pupae to adulthood (Fig. 2); the pupae develop adult structures but fail to enclose and subsequently die (Fig. 3b). At the warmer temperature, the sibs of these same offspring survive pupation but do not display normal adult morphologies. Fully 95% of the Indian dam-by-Canada sire hybrid adults and 44.4% of the Canada dam-by-Indian sire hybrid adults do not form proper elytra (Fig. 3c). These hybrids complete pupation and emerge as adults, but the elytra hang away from the thorax on either side, leaving the beetle vulnerable to desiccation and cannibalism.

## Discussion

We find that hybrid incompatibility manifests early in development as changes in the duration of larval instars and as diminished success in the transition between instars relative to the pure-bred parent populations. Parent populations with similar developmental profiles may nevertheless produce hybrids with disrupted development. The degree and the timing of expression of the larval hybrid inviability are conditional upon the populations crossed, the direction of the cross, and the environment in which the hybrids are raised. Our findings suggest that local adaptation tends to affect the timing of events in early development, which, in turn, plays a significant role in generating hybrid incompatibilities between populations.

We find that the variation among crosses and treatments for growth parameters is not correlated with eventual incompatibility. Crosses whose hybrids prove inviable at the pupal or adult stage tend to have larval growth rates, egg-to-larval viabilities, and asymptotic sizes equal to or exceeding those of the parent populations. The degree of pupal or adult inviability is not correlated with the parameters of larval development. Not including the combinations previously shown to be larval inviable, the larval portion of the beetle's lifespan is unaffected by the novel genomic environment produced by hybridization. While hybrids in a portion of crosses never reach reproductive maturity, we find that the inviabilities are clustered at discrete time points, particularly, at the transitions between larval stages or between the pupal and adult stage. This suggests that the coordinated expression of genes involved in these transitional periods of development is the underlying cause of hybrid incompatibility in this species.

As shown previously (Drury et al. 2011), Indian and Colombian hybrid offspring cease development at the third larval instar, but do not die. They live as third instar larvae for a period as long as the normal adult lifespan. Interestingly, between hatching and the onset of the third instar, they appear to develop normally; when reared at low density, they have the 11th highest *k* and, reared at low density, the 22nd highest *k* (out of the total 28 crosses reared at 34°C). This suggests that discrete stage-dependent developmental errors are being caused by differences between populations in a developmental switch or timing mechanism.

A phenotypically distinct hybrid incompatibility occurs in crosses between the Canadian and Indian populations. Here, larval development proceeds normally to pupation before the hybrid inviability is manifested; the nature of the inviability depends up on the direction of the hybrid cross and the environment in which the hybrids develop. When Canadian sires are mated to Indian dams and the resulting hybrids are reared at 26°C, there is almost complete failure to complete pupation. In contrast, when reared at 34°C, the hybrid offspring progress successfully through the pupal stage, to become free-living adults, all of which have malformed elytra. The reciprocal cross produces the same hybrid phenotypes but they differ in abundance. For the Indian sire-by-Colombian dam offspring reared at 26°C, 60% fail to eclose from pupation; while the other 40% successfully eclose to adults, but incur the elytral deformity. When raised at the higher temperature, the degree of incompatibility diminishes further: 40% of the hybrid adults have malformed elytra, while the other 60% are normal adults. Thus, higher temperature partially rescues hybrid inviability. Those beetles surviving to adulthood, with or without deformed elytra, are fertile and lay viable eggs.

Overall, we find that interpopulation hybrids manifest a variety of hybrid incompatibilities, which vary in degree from partial to complete reciprocal inviability. Furthermore, the expression of hybrid inviability occurs at different but highly specific developmental transitions, depending upon the populations crossed, the direction of the cross, and the temperature at which the hybrid larvae are reared. The among-population variation in hybrid inviability and the population variation in its sensitivity to temperature reveal that pairs of populations can be incompatible in different ways. This suggests that local adaptation affects developmental timing with the result that the developmental incompatibilities between populations have different underlying genetic causes. Although all the developmental differences among hybrids affect the successful progression from one developmental stage to the next, the timing varies with cross, cross direction, and temperature. Until more incompatibilities are mapped to the gene level (e.g., Drury and Wade 2011), it is not clear whether populations are beginning to differentiate in a small number of regulatory genes controlling an iterated set of molts or in a much larger number of genes in a complex regulatory network turned off and on at each larval molt.
